# Comparing the effect of multi-gradient echo and multi-band fMRI during a semantic task

**DOI:** 10.1162/IMAG.a.1043

**Published:** 2025-12-11

**Authors:** Ajay D. Halai, Richard N. Henson, Paola Finoia, Marta M. Correia

**Affiliations:** MRC Cognition and Brain Sciences Unit, University of Cambridge, Cambridge, United Kingdom; Department of Psychiatry, University of Cambridge, Cambridge, United Kingdom

**Keywords:** BOLD fMRI, multi-echo, multi-band, semantic cognition, slice leakage

## Abstract

The blood oxygenation level dependent (BOLD) signal, as measured using functional magnetic resonance imaging (fMRI), is known to vary in sensitivity across the brain due to magnetic susceptibility artefacts. For example, the ventral anterior temporal lobes have been implicated with semantic cognition using convergent methods (i.e., neuropsychology, PET, MEG, brain stimulation) but less so with fMRI using conventional gradient-echo protocols. Of the methods to alleviate this signal loss, “multi-echo” fMRI has gained popularity. Here, additional volumes are collected with a range of echo times (TEs), subsequent combination of which can improve BOLD contrast-to-noise. However, these additional volumes normally require compromising other MR sequence parameters (e.g., longer repetition times, higher in-plane acceleration). One solution is to combine multi-echo with “multi-band” imaging, in which simultaneous acquisition of multiple slices reduces repetition time again. However, it remains unclear how these two modifications independently or interactively affect fMRI sensitivity across the brain, for univariate or multi-variate analyses. In the current study, we used a factorial design in which the number of echoes and/or bands was manipulated to assess how well semantically related activation/multi-voxel patterns can be detected. When comparing the precision with which activations were detected (i.e., average T-statistics), we found that multi-band protocols were beneficial, with no evidence of signal leakage artefacts. When comparing the magnitude of activations, multi-echo protocols increased activations in regions prone to susceptibility artefacts (particularly in the temporal lobes). Both multi-banding and independent component analysis (ICA)-denoising of multi-echo data tended to improve multi-voxel decoding of conditions. However, multi-echo protocols reduced activation magnitude in more central regions, such as the medial temporal lobes, possibly due to the higher in-plane acceleration entailed. Nonetheless, the multi-echo, multi-band protocol is a promising default option for fMRI on most regions, particularly those that suffer from susceptibility artefacts, as well as offering the potential to apply advanced post-processing methods to take advantage of the increased temporal (or spatial) resolution of multi-band protocols and more principled ICA-denoising based on TE dependence of BOLD signals.

## 1 Introduction

Gradient-echo functional magnetic resonance imaging (fMRI) using the blood oxygenation level dependent (BOLD) signal has become the dominant non-invasive tool for studying how the brain operates; however, its ability to detect signal across the whole brain is not homogeneous. This can lead studies to be blind to activation within areas affected by susceptibility artefacts, such as the ventral anterior temporal lobes (ATL) that are important for semantic cognition (see Lambon Ralph et al., 2017 for a review). There have been multiple attempts to overcome these issues with fMRI, and recently multi-echo fMRI has gained popularity (see Kundu et al., 2017 for a review). Typical fMRI studies collect a single echo, which results in sensitivity to a narrow range of T2* values (ideally for maximal sensitivity to BOLD contrast); however, T2* is known to vary across the brain and between participants (Hagberg et al., 2002). Therefore, taking multiple echoes can improve sensitivity by optimally weighting a range of T2* values. This is particularly important for regions with susceptibility artefacts, allowing signal to be detected before it dephases. There have been a number of previous studies comparing multi-echo with typical or modified fMRI protocols, which have argued in favour of multi-echo approaches. However, there are a number of caveats associated with this literature, as discussed below.

Firstly, many studies use only a multi-echo protocol, and compare multi-echo results with those from analysing just one of the echo times (TEs) extracted from that protocol (usually the TE optimal for BOLD, e.g., 25–35 ms for 3T; Amemiya et al., 2019; Bhavsar et al., 2014; Caballero-Gaudes et al., 2019; Cohen & Wang, 2019; Cohen et al., 2017, 2018; Dipasquale et al., 2017; Evans et al., 2015; Fernandez et al., 2017; Gilmore et al., 2022; Halai et al., 2014, 2015; Heunis et al., 2021; Kovářová et al., 2022). However, while this approach has the advantage that the data to be compared are acquired at the same time, the single-echo time series suffer from the MR sequence changes required for multi-echo acquisition, that is, are likely to be inferior to those acquired during a standard (optimised) single-echo sequence. This is because multi-echo acquisition often entails higher in-plane acceleration, reduced k-space sampling, etc., in attempt to reduce repetition times, but which also result in aliasing or noise enhancement (e.g., Deshmane et al., 2012). The single-echo time series extracted from a multi-echo acquisition is, therefore, an unfair baseline from which to determine benefits of multi-echo modifications. Critically, we found only two studies that compared a multi-echo protocol against a separate, typical single-echo protocol (Kirilina et al., 2016; Poser et al., 2006); remaining studies used a multi-band accelerated single-echo protocol (Cohen, Chang, et al., 2021; Cohen, Jagra, Visser, et al., 2021; Cohen, Jagra, Yang, et al., 2021; Cohen, Yang, et al., 2021; Fazal et al., 2023; Lynch et al., 2020) and/or compared different denoising strategies (Lombardo et al., 2016). Overall, the studies found that multi-echo improved functional contrast-to-noise ratio (CNR), particularly in susceptibility-prone regions (Poser et al., 2006), and also showed impaired CNR in deep brain regions (Fazal et al., 2023; Kirilina et al., 2016).

Secondly, another recent advance in echo planar imaging (EPI), often referred to as simultaneous multi-slice or “multi-band” imaging, has made it possible to acquire whole-brain fMRI datasets with spatial or temporal resolution that are several times higher than typical protocols (Moeller et al., 2010; Setsompop et al., 2012). Multi-band imaging has been shown to reduce temporal aliasing of high frequency noise sources, increase statistical power, and improve resting-state network estimation/reliability (Feinberg et al., 2010; Griffanti et al., 2014; Smith et al., 2013). However, fMRI sequences with very high multi-banding acceleration can also impair SNR (e.g., Chen et al., 2015; Demetriou et al., 2018; Risk et al., 2021; Smith et al., 2013) due to a decrease in the level of steady-state longitudinal magnetisation present during imaging at shorter TR and the increase in g-factor noise from the reconstruction of the slice-aliased data (Setsompop et al., 2012). Moreover, while multi-band modifications have mainly been promoted for improved estimation of resting-state connectivity (Smith et al., 2013; Uğurbil et al., 2013), their benefit for task-based fMRI analysis has been less clear (Demetriou et al., 2018; Todd et al., 2017), since low-frequency fMRI noise is unlikely to be correlated (phase-locked) with the task regressors, and so can be removed by high-pass filtering the data. As we are interested in task activations, we will utilise MB to increase the number of volumes (rather than temporal resolution per se). Another potential disadvantage of multi-band acquisition is false positives due to signal leakage across simultaneously excited slices (Todd et al., 2016; Xu et al., 2013). This may be a particular problem for multivariate analyses, whose high sensitivity to patterns across voxels associated with task conditions might reveal “ghost” patterns from leakage to simultaneous slices beyond the slice covering the true pattern. Despite these caveats, studies have combined multi-band with multi-echo imaging, to compensate for the decreased spatial and/or temporal resolution resulting from collecting additional TEs. However, it is difficult to disentangle the effects of echo and band without both elements being manipulated independently.

Thirdly, the criteria used to determine whether multi-echo protocols add benefit vary in the literature. Many studies use rs-fMRI to estimate signal quality through measures such as temporal signal-to-noise ratio (tSNR), although some studies have investigated changes to functional connectivity/networks. In contrast, task-based fMRI studies have focused on CNR in primary sensory cortices (using, e.g., visual checkerboard, finger tapping, breath hold). In this study, we use the semantic network as a test bed, because activity is found across bilateral temporal and frontal lobes, in particular the anterior lateral temporal lobe (ATL; see Lambon Ralph et al., 2017 for a review), which are prone to susceptibility artefacts (Binney et al., 2010; Devlin et al., 2000; Humphreys et al., 2015; Visser, Embleton, et al., 2010).

Here, we examined the effects of protocol both on activation magnitude (e.g., percent signal change difference between two task conditions) and on activation “precision”, which is a noise-normalised measure, where the difference in activation magnitude is divided by an estimate of uncertainty in the estimate of that difference (equivalent to a T-statistic, and comparable with CNR). A priori, we would expect ME protocols to recover signal in regions prone to susceptibility artefacts, resulting in greater effects of task on activation magnitude (which may also translate into higher activation precision too, if noise is unaffected by the protocol). However, most previous studies have reported lower activation magnitude (beta estimates) co-occurring with increases in activation precision (t-values) (Gilmore et al., 2022; Gonzalez-Castillo et al., 2016; Heunis et al., 2021). This is likely to reflect the way that the multiple echoes are combined, since the strategy proposed by Posse et al. (1999) optimises for BOLD CNR, rather than pure BOLD contrast. MB protocols, however, should improve activation precision by virtue of a greater number of volumes and reduced aliasing of high-frequency noise, but should not affect magnitude (since the signal magnitude is independent of how often it is sampled). We tested the reliability of any differences between protocols in terms of T-tests across participants on both activation magnitude and activation precision estimates within each participant.

Another advantage of obtaining multiple echoes arises during post-processing, which leverages on the fact that BOLD signal will show a linear dependency on TE, whereas noise sources do not necessarily show such a dependency. This enables a more automated and principled way of de-noising fMRI data, for example, through independent component analysis (ICA), and projecting out of the data those components that do not show a linear dependence on TE (Kundu et al., 2012, 2013). This is especially helpful for functional connectivity analysis, but we test here on the results of task-based analyses.

Finally, all studies to date have focused on univariate activations for task-based paradigms. This approach is the most widely used method for identifying brain regions activated during a cognitive process, in terms of differences between conditions at each voxel, or after averaging over voxels within a region. In contrast, multivariate methods (such as multivoxel pattern analysis [MVPA]) utilise patterns of activation across many voxels (often irrespective of their mean level of activation) to determine whether those patterns differ between conditions (e.g., Coutanche, 2013; Davis & Poldrack, 2013). Here we estimated the ability of multi-voxel patterns to distinguish (“decode”) two conditions. There is a growing literature suggesting that MVPA can be more powerful than univariate analysis because it exploits variance and covariance between voxels, which is discarded in univariate analyses, and can allow for participant-specific differences in the patterns by analysing derived measures across participants, such as decoding ability (Davis et al., 2014).

In this study, we evaluate the performance of different fMRI protocols to detect both univariate activation and multivariate decoding during a semantic task. The study is unique in four important aspects. First, the “baseline” comparison comes from an independent run using a typical protocol that is optimised for single-band, single-echo fMRI. Second, we manipulate number of echoes and bands independently, resulting in a 2 x 2 factorial design to better separate the effects of multi-echoes and multi-banding (and any potential interaction between these factors). Third, our semantic judgement task is known to activate a network spanning areas that typically have both good and poor image quality. Fourth, we investigate the effects of fMRI protocol on activation magnitude, activation precision, and MVPA. Fifth, we examine any benefits of ICA-denoising of multi-echo data on task-based data, but now with a fairer single-echo comparison. Finally, we also investigated whether multiple bands resulted in false positives due to slice leakage, for both univariate and multivariate analyses.

## 2 Materials and Methods

### 2.1 Participants

We recruited 21 healthy native English speakers (13 females, mean age = 28.9 ± 8.75 years, range 19 to 50 years). All participants were right-handed, had normal or corrected-to-normal vision, and no known neurological disorders (dyslexia, neurodegeneration, etc.). The experiment was approved by the Cambridge Psychology Research Ethics Committee (CPREC).

### 2.2 Stimuli design

**Fig. 1.**
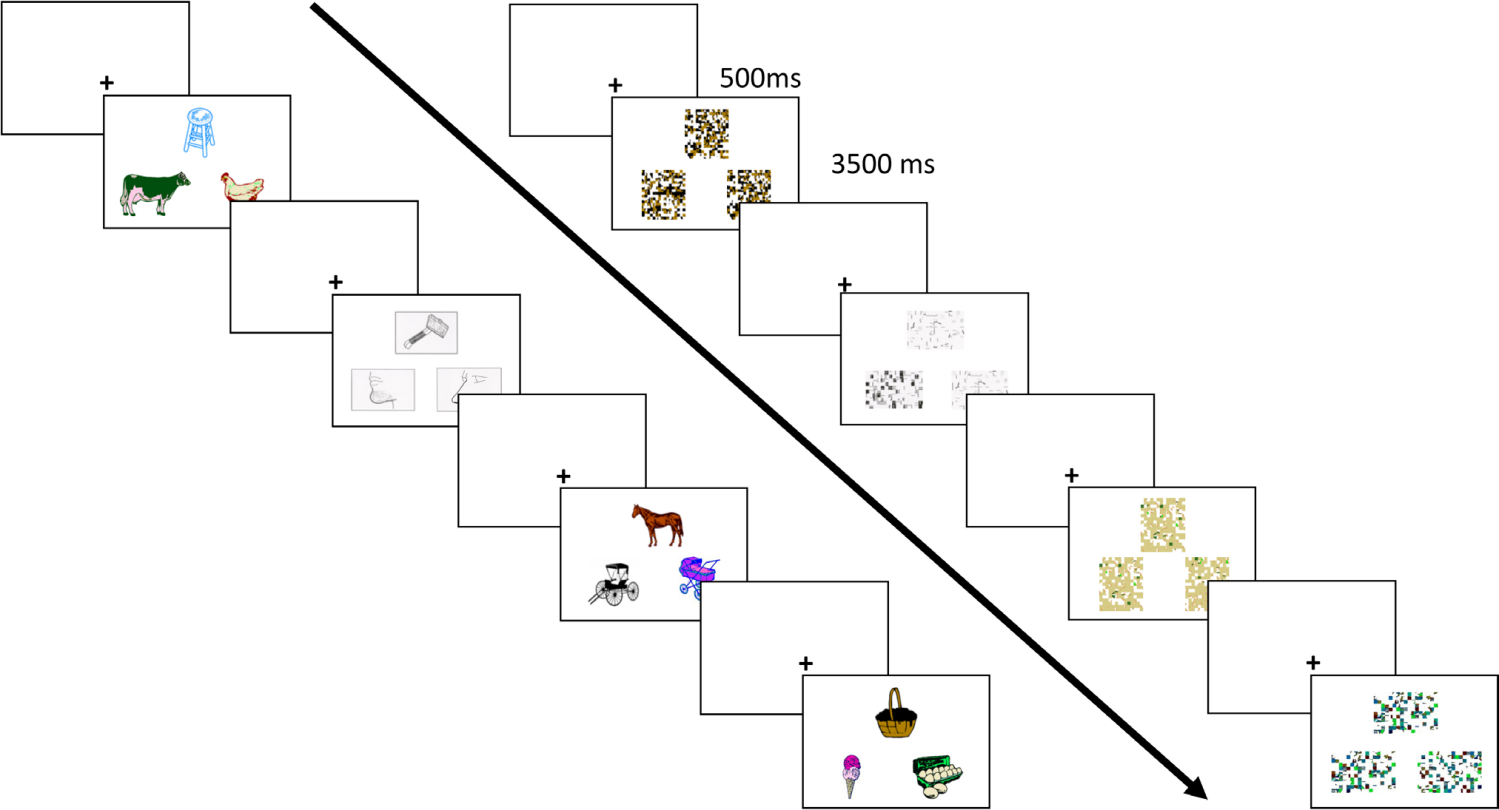
Semantic (left) and control (right) blocks of the triad task. Each block lasted 16 s that included 4 trials, where each trial had a 500 ms fixation and 3500 ms stimulus presentation. Participants were given a probe at the top and were asked to press the left/right button to indicate which of the two pictures below matched the probe.

Participants completed a semantic “triad” task, variants of which have previously been shown to produce robust activation of the ventral anterior temporal lobes using dual-echo fMRI (Jung et al., 2017) and spin-echo fMRI (Humphreys et al., 2015). In each trial, three pictures are presented for a matching task (Fig. 1). In the semantic condition, the participant is required to press a left or right button to indicate which of the two pictures on the bottom of the screen has a semantic relationship with the (probe) picture on the top. For example, if the probe is stool and the options are cow and chicken, one would select the cow. In the control condition, the pictures are scrambled, and the task is a perceptual rather than semantic match, that is, to indicate which bottom image is identical to the top image. The pictures were extracted from the Pyramid and Palm Trees test (Howard & Patterson, 1992) and Camel and Cactus test (Bozeat et al., 2000). EPRIME software was used to display the stimuli and record responses.

We used a block design consisting of 12 replications of 3 types of blocks: semantic (S), control (C), and rest (R). The task started after a 16 s delay after the MR protocol and all subsequent rest blocks were also 16 s. The 36 blocks were ordered S-C-R-C-S-R (x6) (except for the first two participants for whom the order was S-C-R x12). Each block contained 4 trials with the following structure: fixation for 500 ms followed by a triad for 3500 ms, resulting in a total block length of 16 s. The paradigm lasted 592 s in total including rest and was repeated for each fMRI protocol. Accuracy and reaction time were measured for each trial.

We performed a 2 x 2 x 2 ANOVA (condition x multiband x multi-echo) for accuracy and reaction time separately to test for differences in behavioural performance.

### 2.3 Data acquisition

Data were acquired using a 3T Siemens Prisma FIT scanner with a 32-channel RF head coil. The gradient-echo planar imaging (EPI) field of view (FOV) was oriented approximately 30° degrees up from the AC-PC line at the front of the head (to avoid Nyquist ghosting artefacts from the eyes), and full coverage of the temporal lobes was ensured by positioning the FOV at a cost of missing the top of the brain (i.e., superior parietal lobe which is not considered critical region for this particular task).

We matched the four EPI protocols as much as possible across multiple parameters, while manipulating echoes and/or banding. For all EPI protocols, 80 x 80 pixel slices of 3 mm x 3 mm were acquired in a descending, sequential order, with a 0.45 mm gap between slices to minimise cross-talk between imperfect slice excitation profiles (i.e., final voxel size was 3 x 3 x 3.45 mm^3^). A partial Fourier = 7/8th acquisition was used.

The parameters that then differed across protocols are shown in Table 1. For the MB protocols, we used an MB factor of 2, and matched spatial parameters (i.e., same slice thickness), resulting in better temporal resolution, that is, halved TR. The flip angle was optimised for each TR. For the ME protocols, we acquired three echoes (13.00, 25.85, and 38.70 ms), and used a GRAPPA acceleration of 3, resulting in a slightly faster acquisition, so we increased the number of slices from 28 to 30 in order to better match the TR. We decided to use GRAPPA = 3 instead of GRAPPA = 2, as during piloting we found that the shortest TE achieved with the latter was higher than previous studies (15 ms).

**Table 1.** Parameters for the four EPI protocols compared in a 2 x 2 factorial design.

	Single Echo Single Band (SESB)	Single Echo Multi-Band (SEMB)	Multi-Echo Single Band (MESB)	Multi-Echo Multi-Band (MEMB)
Echoes	1	1	3	3
Multi-band factor	1	2	1	2
TR (ms)	2000	1000	2000	1000
TE 1 (ms)	30.00	30.00	13.00	13.00
TE 2 (ms)	-	-	25.85	25.85
TE 3 (ms)	-	-	38.70	38.70
GRAPPA acceleration	off	off	3	3
Flip angle (degrees)	78	62	78	62
Slices	28	28	30	30
Z-coverage (mm)	96.6	96.6	103.5	103.5

Repetition time (TR); echo time (TE).

An MPRAGE structural image was acquired with the following parameters: TR = 2250 ms, TE = 3.02 ms, TI = 900 ms, GRAPPA = 2, FOV = 256 mm × 256 mm × 192 mm, voxel size = 1 mm^3^, flip angle (FA) = 9°.

### 2.4 Data analysis

Data and code have been made publicly available at https://github.com/AjayHalai/Sensitivity_of_MEMB.

#### 2.4.1 Preprocessing

For reproducibility and data sharing, we converted all DICOMs to BIDS format (K. J. Gorgolewski et al., 2016) using the *HeuDiConv* tool (v0.9.0; Halchenko et al., 2020) and used an fMRIprep (v22.0.0) (Esteban et al., 2019; K. Gorgolewski et al., 2011) singularity container to process all imaging data. The only exception was for the multi-echo datasets, which were re-processed using the *tedana* tool (v0.0.11) (DuPre et al., 2021) where we took the minimally processed data from the fMRIprep pipeline (details below).

The T1-weighted (T1w) image was corrected for intensity non-uniformity with *N4BiasFieldCorrection* (Tustison et al., 2010), distributed with *ANTs* (v2.3.3) (Avants et al., 2008), and used as T1w reference throughout the workflow. The T1w reference was then skull stripped with an Nipype implementation of the *antsBrainExtraction.sh* workflow (from *ANTs*), using OASIS30ANTs as target template. Brain tissue segmentation of cerebrospinal fluid (CSF), white matter (WM), and grey matter (GM) was performed on the brain-extracted T1w using *fast* (FSL v6.0.5.1, Zhang et al., 2001). Volume-based spatial normalisation to standard space (TemplateFlow ID: MNI152NLin2009cAsym; Fonov et al., 2009) was performed through nonlinear registration with *antsRegistration* (from *ANTs*), using brain-extracted versions of both T1w and the T1w template.

For each of the functional runs, the following pre-processing was performed. First, a BOLD reference volume (from the shortest echo) and its skull-stripped version were generated using a custom methodology in fMRIPrep. Head-motion parameters with respect to the BOLD reference (transformation matrices, and six corresponding rotation and translation parameters) are estimated before any spatiotemporal filtering using *mcflirt* (Jenkinson et al., 2002). BOLD runs were slice time corrected to the middle slice using *3dTshift* from *AFNI* (Cox & Hyde, 1997). The slice time-corrected data were corrected for head motion (referred to as preprocessed BOLD). The BOLD reference was then co-registered to the T1w reference using mri_coreg (FreeSurfer) followed by *flirt* (Jenkinson & Smith, 2001) with the boundary-based registration (Greve & Fischl, 2009) cost function with six degrees of freedom. Several additional confounding time series were calculated (i.e., framewise displacement (FD), DVARS and three region-wise global signals, anatomical/temporal component-based noise correction), but we only used the six motion (translation and rotation) parameters in this study, therefore, the other metrics are not described further.

For multi-echo data, we used *tedana* to reprocess the minimally pre-processed BOLD data (slice time and motion corrected) from the fMRIprep pipeline in order to obtain the ICA-denoised time series. A whole-brain mask derived from the T1 was used to define brain space, to which a two-stage adaptive masking procedure was implemented. First, a liberal mask (including voxels with good data in at least the first echo) was used for optimal combination, T2*/S0 estimation, and denoising, while a more conservative mask (restricted to voxels with good data in at least the first three echoes) was used for the component classification procedure. A mono-exponential model was fit to the data at each voxel using nonlinear model fitting in order to estimate T2* and S0 maps, using T2*/S0 estimates from a log-linear fit as initial values. For each voxel, the value from the adaptive mask was used to determine which echoes would be used to estimate T2* and S0. In cases of model fit failure, T2*/S0 estimates from the log-linear fit were retained instead. Multi-echo data were then optimally combined using the T2* combination method (Posse et al., 1999). Principal component analysis based on the PCA component estimation with a moving average (stationary Gaussian) process (Li et al., 2007) was applied to the optimally combined data for dimensionality reduction. Next, an independent component analysis (ICA) was then used to decompose the dimensionally reduced dataset from the PCA. Kappa (kappa) and Rho (rho) values were calculated as measures of TE-dependence and TE-independence, respectively for both PCA and ICA reduced datasets. Finally, component selection was performed to identify BOLD (TE-dependent), non-BOLD (TE-independent), and uncertain (low-variance) components using the Kundu decision tree (v2.5; Kundu et al., 2013). This workflow used numpy (Walt et al., 2011), scipy (Jones et al., 2001), pandas (McKinney, 2010), scikit-learn (Pedregosa et al., 2011), nilearn, and nibabel (Brett et al., 2019). This workflow also used the Dice similarity index (Dice, 1945; Sørensen, 1948). We retained two final time series from this pipeline: optimally combined T2* (t2star) and ICA-denoised (ICA-denoised).

The BOLD time series from all protocols were resampled into standard space, generating a pre-processed BOLD run in MNI152NLin2009cAsym space. All resamplings can be performed with a single interpolation step by composing all the pertinent transformations (i.e., head-motion transform matrices and co-registrations to anatomical and output spaces). Volumetric resampling was performed using *antsApplyTransforms* (*ANTs*), configured with Lanczos interpolation to minimise the smoothing effects of other kernels (Lanczos, 1964). For voxel-wise analysis, all images in MNI space were smoothed using 8 mm^3^ kernel in SPM12 in order to account for imperfect spatial registration and to maintain the same level of smoothing as the typical single-echo sequence. For all ROI analyses reported, we used unsmoothed data.

We used the six rigid motion parameters as a measure of image quality to determine whether there was a significant difference between each protocol. We performed an ANOVA separately for each motion parameter (x, y, z translation and pitch, roll, yaw rotation).

#### 2.4.2 1st level (within-participant) GLM

Statistical analysis was carried out using used SPM12 (v) in MATLAB r2019a to create 1st level general linear models (GLM) for each protocol of interest. To be explicit, we had a 2 x 2 factorial design (SESB, SEMB, MESB, MEMB) but we also investigated specific effects for (1) ICA-denoised data (with “dn” suffix) and (2) a reduced multi-band dataset by extracting only odd volumes to match the number of time points in the non-accelerated protocol (with “odd” suffix), resulting in SEMBodd, MESBdn, MEMBdn, and MEMBodd. For each participant and protocol, we created a contrast to identify regions associated with semantic processing (semantic > control). Additionally for the decoding analysis, we modelled each block using separate regressors (12 semantic and 12 control) to obtain beta images per block.

The following additional parameters were applied to all models: six motion parameters as regressors of no interest; micro-time resolution set to number of slices, and micro-time onset to number of slices/2; high-pass filter of 128 s; AR(1) model to account for serial correlations; and we turned off SPMs automated mask threshold and supplied a brain mask extracted from individual T1.

#### 2.4.3 2nd level (between-participant) analysis

##### 2.4.3.1 Region of interest (ROI) analysis

###### 2.4.3.1.1 Univariate analysis

We had an a priori hypothesis that the semantic network would be activated during the semantic > control contrast. We defined regions of interest (ROIs) as spheres of 8 mm radius centred on the peak coordinates from a large-scale spin-echo fMRI study that incorporates multi-model (visual and auditory) language tasks (Humphreys et al., 2015); however, given the large number of ROIs, here we only inspected those that had >50% overlap with the overall main effect of condition in the present study (an F-test across all protocols). We used a custom MATALB script to extract ROI information built on SPM tools (“*roi_extract.m”* from https://github.com/MRC-CBU/riksneurotools/tree/master/Util). For each ROI, we extracted two metrics: (1) activation magnitude, that is, the difference in GLM parameter estimates (“betas”) for semantic minus control blocks and (2) activation precision, that is, the t-statistic for that difference, that is, the activation magnitude scaled by an estimate of the uncertainty of that difference, which also depends on the noise in the data. Then to determine the reliability of these two metrics, we performed planned t-tests across participants to compare protocol choices, that is: ME > SE (main effect of echo, i.e. [MEMB+MESB] – [SEMB+SESB]), MB > SB (main effect of band, i.e., [MEMB+SEMB] – [MESB+SESB]), the interaction between echo and band (i.e., [MEMB-SEMB] – [MESB-SESB]), MEdn > ME (effect of denoising, i.e. [MEdnMB+MEdnSB] – [MEMB+MESB]), and MBodd > SB (effect of sampling rate, i.e. [MEMBodd + SEMBodd] – [MESB+SESB]). Results are reported using at p < 0.05 (uncorrected) and p < 0.05 few corrected using permutation testing of the maximal T-statistic across all tests (N = 2^n^, where n = 16).

We focused on two univariate effects: the effect of MR protocol on (1) activation magnitude (BOLD signal change) and (2) activation precision (reliability of BOLD change, analogous to CNR). The former was tested by comparing across MRI protocols the difference between the 1st-level betas for semantic > control conditions for each participant; the latter was tested by comparing the 1st-level T-statistic for this contrast instead (i.e., activation magnitude normalised by estimate of residual error across scans).

###### 2.4.3.1.2 Decoding analysis

We also tested whether the protocols affected multivariate effects, namely decoding of condition using multivoxel pattern analysis (MVPA). For this, we extracted beta values within each ROI for each task block, which resulted in a 24 (12 semantic and 12 control blocks) x 280 (voxels per ROI) matrix. From this, we estimated the dissimilarity (using a cosine measure) between the patterns for every pair of blocks, and then averaged those according to whether they were within the same condition or from different conditions. We then subtracted the mean between-condition dissimilarity from the mean within-condition dissimilarity (which should be positive if decoding is possible), and used a non-parametric t-test to test whether this decoding metric differed for the contrasts across MR protocols described in the univariate section above. Decoding should be sensitive to factors that affect activation magnitude and activation precision (at individual voxels), and is potentially more sensitive than univariate analyses (Davis et al., 2014).

##### 2.4.3.2 Whole-brain analysis

In order to determine whether any effects were observed outside the a priori semantic network ROIs, we calculated whole-brain effects using a 2 x 2 ANOVA to test for main effects of echoes and band. We used a custom MATLAB script built on SPM tools (“*batch_spm_anova.m”* from https://github.com/MRC-CBU/riksneurotools/tree/master/GLM) and extracted the following group-level contrasts for semantics: (1) ME>SE; (2) SE>ME; (3) MB>SB; (4) SB>MB; and (5) the overall main effect of all protocols together. We applied a voxel-height threshold of p < 0.001 uncorrected to define clusters, and then used a cluster-wise Family-Wise-Error corrected p < 0.05 for statistical inference.

##### 2.4.3.3 Slice leakage analysis

To evaluate the potential impact of slice-leakage artefacts on this dataset, candidate artefact locations (artefact voxels) were identified for the MEMB and SEMB protocols, using a modified version of the MATLAB scripts developed for McNabb et al. (2020) (https://github.com/DrMichaelLindner/MAP4SL) (implemented in MATLAB 2020b). We conducted two types of analyses: (1) comparing univariate t-values and (2) comparing multivariate decoding.

**Fig. 2.**
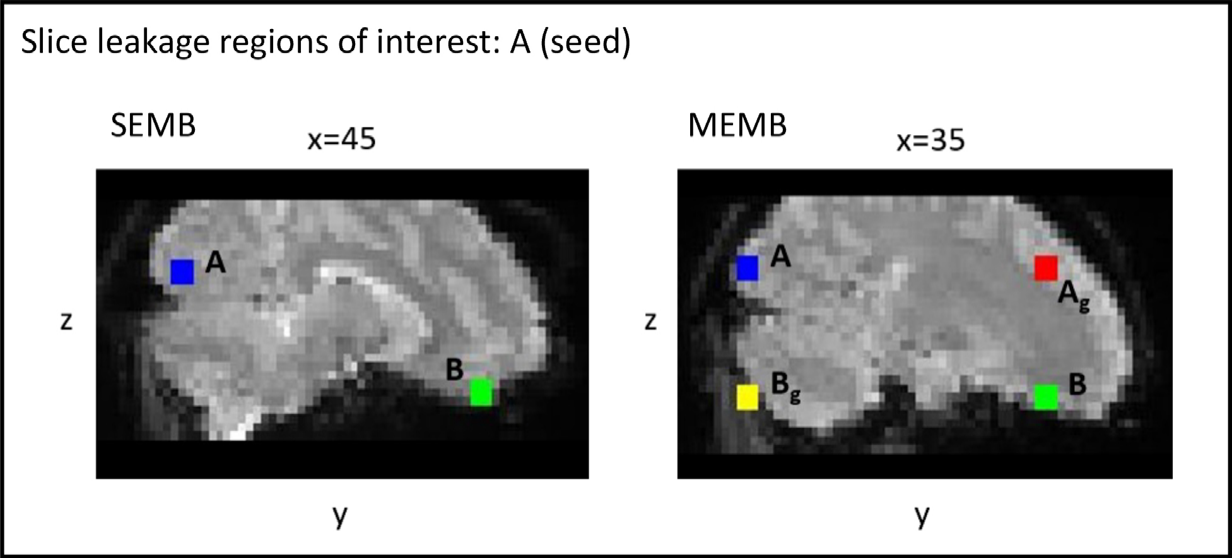
Example location of seed and artefact ROIs. Seed ROI A [blue] and expected artefact locations for an individual participant. ROI B [green] represents the artefact location due to the multi-band protocol (phase shift of FOV/2), while ROI Ag [red] and Bg [yellow] represent the artefact locations due to in-plane acceleration (GRAPPA factor of 3). ROIs are shown for single-echo and multi-echo data.

For the univariate analysis, we created GLMs on the pre-processed native EPI data (i.e., no normalisation or smoothing) and identified the voxel corresponding to the maximum t-value for each participant, separately for the SEMB and the MEMB data (seed voxels). For the SEMB protocol, GRAPPA was not used for in-plane acceleration and, therefore, only one alias location was expected per slice due to the MB protocol, with a phase shift of FOV/2 (Fig. 2, left). For the MEMB protocol, there were two alias locations per slice due to multi-banding and in-plane acceleration (Fig. 2, right). An ROI of 3 x 3 x 3 voxels was then defined around each seed and corresponding artefact locations from which the mean t-value was calculated for each ROI. The seed ROIs are labelled “A” and the candidate artefact location due to multi-band is labelled “B”. “Ag” represents the expected artefact location for ROI “A” due to GRAPPA, and “Bg” is the equivalent artefact location for ROI “B”.

For the multivariate analysis, we create GLMs on the EPI data in MNI space (but no smoothing), in order to have a greater number of voxels per ROI. The left vATL was used as the seed as this area showed successful decoding and is a key area of interest for the paradigm (see results in Table 2). Artefact locations were created in the same way as described above for the univariate analysis (i.e., in native EPI space), but all final ROIs were projected to MNI space and fixed as a sphere with 8 mm radius.

**Table 2.** T-tests for the planned contrasts using semantic network regions of interest (ROI) for activation magnitude (contrast of betas), activation precision (statistical T-values), and decoding (cosine dissimilarity).

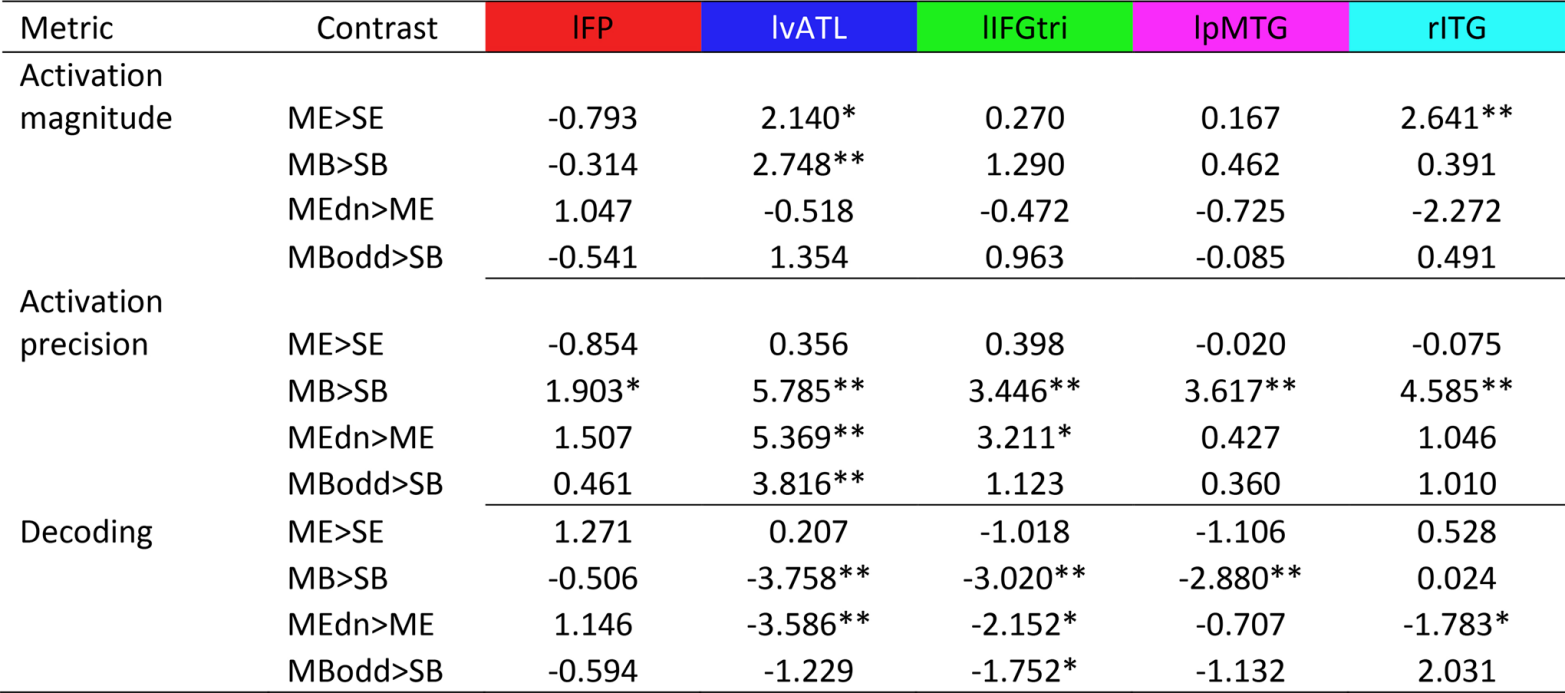

*Uncorrected p < 0.05, **FWE corrected p < 0.05 using permutation of maximal T-statistic across 20 tests.

Inferior frontal pole (IFP), left ventral anterior temporal lobe (lvATL), left inferior frontal gyrus pars triangularis (lIFGtri), left posterior middle temporal gyrus (lpMTG), right inferior temporal gyrus (rITG), multi-echo (ME), single-echo (SE), multi-echo ICA-denoised (MEdn), multi-band odd volumes (MBodd).

To assess whether false-positive activations/decoding due to slice leakage could be observed in the multi-band data, the corresponding single-band data (SESB and MESB, respectively) were used as controls since no aliasing artefacts are expected. For this purpose, we generated seed and artefact ROIs (as defined for SEMB) in the SESB data and extracted t-values/dissimilarity. Similarly, the seed and artefact ROIs generated for the MEMB data were used to extract t-values/dissimilarity from the MESB data. In addition, we compared SESB and MEMB to determine whether there was an adverse effect of in-plane aliasing at Ag and Bg locations.

## 3 Results

### 3.1 Behavioural data and image quality

We identified four participants who had poor behavioural performance (2SD away from group mean, where mean accuracy discarded subjects for all conditions <73% across all runs), and, therefore, removed them from further analysis. We also excluded an additional participant who had poor EPI coverage of the temporal lobes. The final sample of 16 participants had good and comparable performance (Supplementary Materials 1). A 2 (condition) x 2 (SE/ME) x 2 (MB/SB) ANOVA on task accuracy showed a main effect of condition (F(1,15) = 131.086, p < 0.001), with the semantic task (S) having worse performance (M = 78.46 [SD = 6.37]%) than the control task (C) (M = 94.03 [SD = 7.13]%), as expected from previous studies using this paradigm. Importantly, there were no other significant effects, that is, no evidence that performance differed for the different MR protocols (i.e., SE/ME or SB/MB). For RTs, the same ANOVA did reveal a significant three-way interaction (F(1,15) = 6.566, p = 0.022); however, follow-on ANOVAs broken down by one of the factors showed no significant effects, so this three-way interaction is difficult to interpret further and could be a false positive given the number of ANOVA effects tested.

The analysis to compare image quality (based on six rigid motion parameters) revealed no significant differences between protocols. The mean [STD] absolute translation x, y, z was 0.3930 [0.2680], 0.5538 [0.5866], 0.6166 [0.6424], and 0.5519 [0.4570] mm for SESB, SEMB, MESB, and MEMB, respectively. The mean [STD], absolute rotation pitch, roll, yaw was 0.0067 [0.0052], 0.0073 [0.0081], 0.0065 [0.0052], and 0.0064 [0.0035] radians for SESB, SEMB, MESB, and MEMB, respectively.

### 3.2 ROI results

**Fig. 3.**
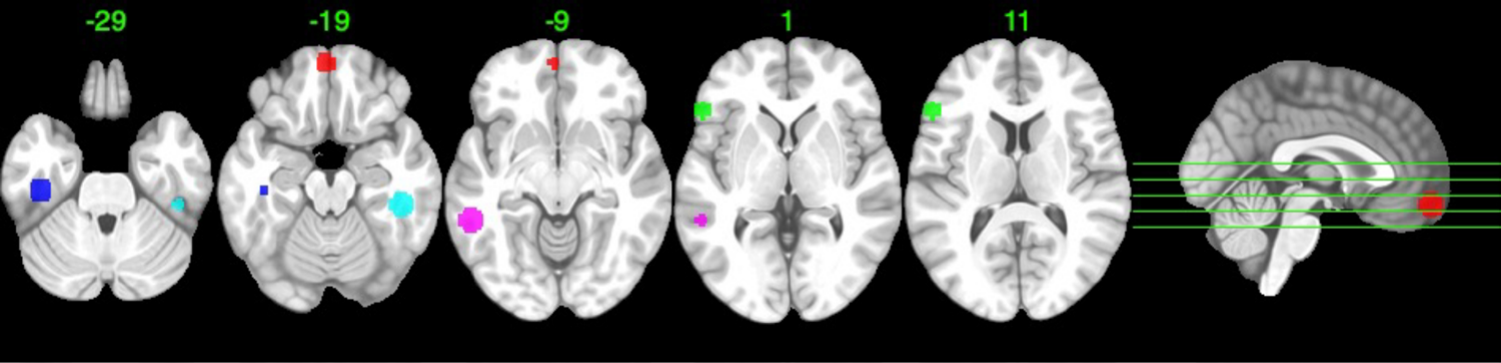
Semantic network regions of interest (ROI; 8 mm spheres) defined using Humphreys et al. (2015) and selecting those that overlap with overall main effect of semantics in the current study. Inferior frontal pole (red), left ventral anterior temporal lobe (blue), left inferior frontal gyrus pars triangularis (green), left posterior middle temporal gyrus (purple), right inferior temporal gyrus (cyan).

The ROIs from Humphreys et al. (2015) that overlapped with significant clusters for the contrast of semantic versus control conditions, averaged across MR protocols, are shown in Figure 3. These included left ventral anterior temporal lobe (vATL), right inferior temporal gyrus (ITG), left inferior frontal gyrus (IFG), left posterior middle temporal gyrus (pMTG), and left frontal pole (FP).

The planned contrasts of interest and resultant statistical p-values are shown in Table 2 (see Supplementary Materials 1 for an extended table of comparisons, including reverse contrasts). For activation magnitude (contrasts on the mean across participants of the difference between betas from S vs. C conditions), ME was significantly higher than SE in right ITG and trending for left vATL. This would be expected if these regions suffer from susceptibility artefacts, causing greater “drop-out” of signal for the SE with non-optimal TE for those regions. For MB, we also found a significant benefit over SB in the left vATL. The ICA-denoising (MEdn) did not improve on the basic ME data. The interaction between echo and band was not significant in any ROI. We show results across a wider ROI network in Supplementary Materials 1.

For activation precision (comparisons across participants of their T-statistics), there were advantages for MB versus SB for all five ROIs, but no differences for ME versus SE. The former main effect of MB is expected from the greater sampling rate (i.e., shorter TR), resulting in more effective degrees of freedom in the data and less aliasing of low-frequency noise. As expected, when removing one half of the (even) volumes (MBodd), this advantage disappeared for most regions, though an MB advantage remained in lvATL. Though there was no evidence for a basic advantage of ME versus SE, once ICA-denoising was applied (enabled by having more than one TE), the activation precision increased (for MEdn vs. ME) in two of the ROIs, suggesting that noise sources are successfully being removed. We did not find any significant results for reverse comparisons for these ROIs (see Supplementary Materials 1 for detailed results).

Finally, the decoding results were very similar to the activation precision results, with no evidence for a benefit for ME over SE protocols, but superior decoding for MB over SB protocols in the left vATL, left IFGtri, and left pMTG. Similarly, ICA-denoising improved data decoding effects in the left vATL, left IFGtri, and right ITG. The matched sampling MB data (MBodd) marginally outperformed the SB protocols in the left IFGtri only. As above, the reverse comparisons showed fewer differences, with an SB advantage observed over the reduced MB time series only in the rITG (p = 0.030).

### 3.3 Whole-brain results

**Fig. 4.**
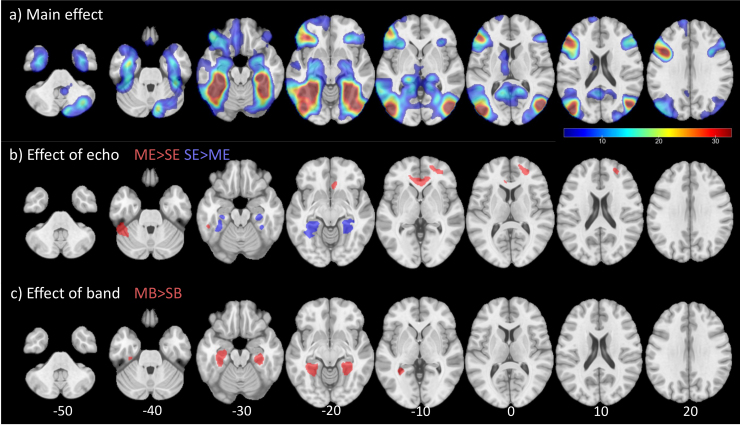
Whole-brain analysis comparing activation magnitude (contrast betas) for semantic > control in a 2 x 2 ANOVA manipulating echo and band fMRI protocols. (a) Average effect across all protocols [t-value 3.28–32.8]; (b) directed effects of echo (ME>SE [red] and SE>ME [blue]); and (c) directed effects of band (MB>SB [red]; SB>MB not significant).

For activation magnitude, Figure 4a shows whole-brain results for the semantic > control contrast, averaged across all protocols (the results for individual protocols are provided in Supplementary Materials 3). Activations are largely bilateral, and include the core semantic regions in the temporal and frontal cortices. Results for the main effects of ME and of MB in the 2 x 2 ANOVA are shown in Figure 4b and c, with peak information summarised in Supplementary Materials 4. The main effect of echo identified three clusters with greater activation magnitude for ME than SE: (1) left inferior temporal/fusiform cortex, (2) left anterior cingulate gyrus, and (3) right frontal pole. By contrast, clusters in bilateral medial temporal fusiform, parahippocampal, and hippocampal regions showed the opposite effect of greater magnitude for SE than for ME. Finally, the main effect of band revealed greater activation magnitude for MB than for SB in similar bilateral medial temporal structures such as fusiform cortex, with no significant clusters for the reverse contrast. No voxels survived correction for the ME-by-MB interaction. There is a suggestion in the literature that smoothing may not be necessary or optimal for ME data, due to the removal of noise by averaging echoes over time rather than space (Gonzalez-Castillo et al., 2016; Gilmore et al., 2022; Ramot et al., 2020). However, other types of noise are not addressed by temporal averaging such as imperfect spatial registration across individuals. For transparency, we reanalysed the data using three levels of smoothing (none, 3 mm, and 8 mm FWHM) and report results in Supplementary Materials 5. We note that results are qualitatively similar and, therefore, not discussed further in the main manuscript.

**Fig. 5.**
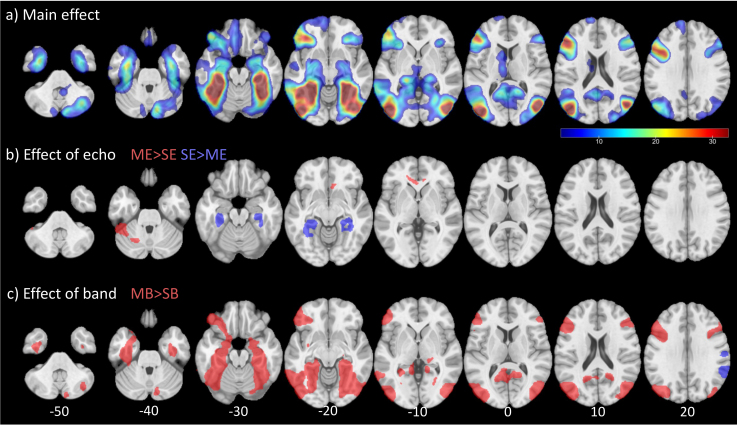
Whole-brain analysis comparing activation precision (statistical t-values) during semantic activation (semantic > control) in a 2 x 2 ANOVA manipulating echo and band fMRI protocols. (a) Overall main effect of all protocols [t-value 3.28–32.8]; (b) directed effects of echo (ME>SE [red] and SE>ME [blue]); and (c) directed effects of band (MB>SB [red] and SB>MB [blue]).

The results using activation precision (Fig. 5) were largely similar to those for activation magnitude. The main noticeable difference was that MB was significantly better than SB in almost all parts of the semantic network, including extending into the ventral anterior temporal fusiform cortex.

We also ran a 2 x 2 ANOVA on the multi-echo data only to test the effect of ICA-denoising and whether this interacted with multi-band. We observed only one cluster surviving correction for activation magnitude, where the main effect of band (MB>SB) identified left posterior fusiform cortex clusters extending up into the right calcarine sulcus (Supplementary Materials 6a). Differences in activation precision were similar to those shown in Figure 5, where the MB>SB contrast showed clusters across the semantic network (Supplementary Materials 6b). Overall, this analysis replicated results showing that MB offers improvements over SB across the entire semantic network; in contrast ICA-denoising did not show specific improvements but was not significantly worse than when not using ICA-denoising.

### 3.4 Slice leakage results

Supplementary Material 7(i) shows the mean t-value per participant for the two seeds (A and B) versus their potential leakage locations for the single-echo protocols, and Supplementary Material 7(ii) shows the data for the multi-echo protocols. For both univariate and multivariate analyses, we used paired t-tests to compare the mean t-values/dissimilarity between corresponding locations for single-echo protocols (t_A,SEMB_ – t_A,SESB_ and t_B,SEMB_ – t_B,SESB_), and for multi-echo protocols (t_A,MEMB_ – t_A,MESB_, t_B,MEMB_ – t_B,MESB_, t_Ag,MEMB_ – t_Ag,MESB_, and t_Bg,MEMB_ – t_Bg,MESB_).

For the univariate analysis, the difference for Seed A was significant between SEMB and SESB (t = 5.817, df = 20, p < 0.001) and also between MEMB and MESB (t = 4.405, df = 20, p < 0.001), reiterating the previous results where MB results in higher t-values than SB protocols. For the artefact locations, no comparison was significant, with the exception of that for seed B in the multi-echo data, where the mean t-values were found to be significantly greater for MESB than for MEMB (t = -2.096, df = 20, p = 0.048). However, the directionality is opposite to what we might expect for slice-leakage artefacts, and the comparison does not survive correction for multiple comparisons.

For the multivariate analysis, the difference for Seed A was significant between SEMB and SESB (t = 3.295, df = 20, p < 0.005), but not between MEMB and MESB (t = 0.2488, df = 20, p < 0.8069). For the artefact locations, no comparison was significant. Therefore, overall the results suggest no evidence of slice-leakage effects for univariate or multivariate analysis in the current dataset.

In addition, we compared SESB and MEMB at locations Ag and Bg to determine whether there was an adverse effect of in-plane aliasing; however, we found no significant differences (see Supplementary Material 7(iii); Ag p = 0.09 and Bg p = 0.479).

## 4 Discussion

Gradient-echo BOLD sensitivity varies across the brain. In particular, the presence of air cavities near the front and lateral sides of the head results in magnetic susceptibility artefacts (signal loss and distortions). Many methods have tried to improve signal detection in such areas while retaining sufficient sensitivity to the rest of the brain, and this study provided a unique opportunity to systematically compare a typical imaging protocol with multi-echo and multi-band modifications. When comparing the precision with which activations were detected during a semantic task (i.e., average T-statistics), we found that multi-band protocols were generally beneficial, with no evidence of signal leakage artefacts, at least for a multi-band factor of 2. This increased precision is to be expected given the greater number of volumes and hence additional degrees of freedom with which to estimate activation. Also as expected, when comparing the magnitude of activations, multi-echo protocols increased activations in regions prone to susceptibility artefacts (specifically the anterior temporal lobes, ATLs), and the addition of ICA-denoising (enabled by measuring multiple echoes) further improved the precision of those activations. Both multi-banding and ICA-denoising of ME data also tended to improve multi-voxel decoding of experimental conditions. However, multi-echo protocols reduced activation magnitude in more central regions, such as the medial temporal lobes (MTLs), presumably due to the higher in-plane acceleration (GRAPPA) required to record multiple echoes.

### 4.1 Advantages and disadvantages of multi-echo protocols

In the current study, we used task-based fMRI to probe semantic cognition because the semantic network includes regions affected by susceptibility artefacts, such as the anterior temporal lobes (ATLs; Lambon Ralph et al., 2017). Detecting fMRI activation in the ventral and lateral ATLs has been challenging, due to a number of factors (reviewed in Visser, Jefferies, et al., 2010), the most important of which is likely to be signal loss when using conventional echo times (TEs) of ~30–35 ms. As noted in the Introduction, modified protocols have been successful at detecting activity using spin-echo fMRI (Binney et al., 2010; Humphreys et al., 2015; Visser, Embleton, et al., 2010) and dual gradient-echo fMRI (Halai et al., 2014, 2015). In the current study, all protocols were able to detect robust activations in bilateral ventral ATL as well as inferior frontal regions. Nonetheless, we demonstrated that certain modifications can lead to further improvements.

A priori, we expected multi-echo to improve activation magnitude in regions prone to susceptibility artefacts by recovering signal drop out, compared with single-echo protocols. In our ROI analysis, we observed such a benefit in the left ATL and right ITG for activation magnitude. Interestingly, this increased signal was not accompanied by higher T-statistics (activation precision)—nor better multivoxel decoding—in these ROIs, suggesting that the ME protocol also increased noise in these regions (e.g., due to the in-plane acceleration required to reduce echo train length). Nonetheless, when leveraging the multiple echoes to improve ICA-denoising, these ROIs did now show increased activation precision and multivoxel decoding (see Supplementary Materials 2).

Signal recovery in areas near susceptible artefacts is expected with multi-echo protocols because the shorter TEs are better able to capture signal before it dephases. This is in line with current language literature using similar modified protocols (i.e., Halai et al., 2014; Jung et al., 2017). When using a whole-brain search, we saw increased activation magnitude and precision in a few other clusters, most notably left posterior inferior temporal gyrus, which is also prone to susceptibility artefacts. Improvements were also seen in left anterior cingulate gyrus and right frontal pole, likely due to increased BOLD sensitivity through optimally combining multiple echoes (better estimation of T2*), suggesting that improvements are not restricted to regions normally associated with susceptibility artefacts. Importantly, we also observed a significant reduction in both activation magnitude and activation precision in more central brain regions, such as the MTL. This is most likely due to lower tSNR in brain regions that are further from the receiver coils, which is exaggerated when using higher g-factors for in-plane acceleration (Kirilina et al., 2016; though Fazal et al., 2023, also reported reduced sensitivity in visual and anterior cingulate regions during a Stroop task for multi-echo multi-band compared with multi-band only).

Note that the precise effects of multiple versus single echoes on activation magnitude and precision are likely to depend on how the multiple echoes are combined (weighted), and how they relate to the optimal TE for a given brain region. Here we weighted the data for each echo according to the default tedana options, which optimises for CNR (Posse, 2012). Previous studies using similar weighting have actually shown reduced activation magnitudes (Gilmore et al., 2022; Gonzalez-Castillo et al., 2016; Heunis et al., 2021). We did not see this decrease in any of our ROIs (perhaps because many are prone to signal drop out), but as noted above, we did see this in some other brain regions in our whole-brain analysis, such as MTL (Supplementary Materials 4). As stated earlier, we suspect that such signal reductions reflect the increased in-plane acceleration relative to our standard single-echo sequence, but this explanation cannot apply to studies that compared results when a single echo is compared with a weighted combination of echoes from the same multi-echo MR sequence. In any case, future work could explore whether these magnitude reductions could be prevented by some other weighting of TEs for such regions.

Finally, we hypothesised that removing noise from the time series should improve activation precision and decoding (but not affect activation magnitude, unless signal is removed by mistake). MR physics dictates that there is a positive linear relationship between TE and BOLD signal amplitude, and this fact is used by automated techniques that deploy ICA and machine learning to automatically separate components likely to be signal from those likely to be noise, such that noise components can then be projected out of the data (DuPre et al., 2021; Kundu et al., 2012, 2013). Many studies have demonstrated the benefit of this “ME-ICA” approach for resting-state fMRI connectivity (e.g., Cohen, Yang, et al., 2021; Lombardo et al., 2016; Lynch et al., 2020); few however have demonstrated this benefit in task-based fMRI. Here we also failed to find any clusters in our whole-brain analysis that survived correction for the main effect of ICA-denoising (for ME protocols averaged across SB/MB; Supplementary Materials 6). This suggests that effects of ICA-denoising are generally weak in task-based analyses, given that noise is rarely correlated with the task design. Nonetheless, we did find evidence that ICA-denoising improves task-related activation precision and decoding when using more sensitive tests within some of our a priori ROIs. These improvements presumably reflect a reduction in overall residual error (noise). Interestingly, one ROI (right ITG) actually showed a reduction in activation magnitude after ICA-denoising (see Supplementary Materials 2), suggesting that some signal of interest may have been removed by mistake. In sum, we propose that automated ICA-denoising methods that use the TE dependence of BOLD enabled by ME protocols can improve task-based statistics, but only modestly, and the risk of also removing signal should be kept in mind.

### 4.2 Advantages of multi-band data

For the multi-band modification, we expected improvements to activation precision owing to having more data and less aliasing of high-frequency fMRI noise, without any impact on activation magnitude. There is some evidence that multi-band protocols can impair sensitivity when combined with in-plane acceleration or reduced k-space sampling (e.g., Chen et al., 2015), but here we were careful to match these to the single-band protocols (apart from the flip angle, which was reduced so as to maximise BOLD sensitivity for the corresponding TR). Multi-band modifications have mainly been promoted for improved estimation of resting-state connectivity (Smith et al., 2013; Uğurbil et al., 2013); their benefit for task-based fMRI analysis has been less clear (Demetriou et al., 2018; Todd et al., 2017), since the reduced low-frequency noise is unlikely to be correlated (phase-locked) with the task regressors, and so can be removed by standard high-pass filtering. Our data show that even a modest acceleration of MB factor (x2) can lead to consistent benefits in activation precision across all a priori ROIs (Table 2). It also improved multivoxel decoding in many of them. However, we note that higher MB accelerations (>4) have been associated with noise amplification or other reductions in signal quality (e.g., Demetriou et al., 2018; Risk et al., 2021; Todd et al., 2016). Therefore, researchers using such high MB factors, such as those in protocols developed for the Human Connectome Project (HCP), should be aware that task-based results might be negatively impacted, particularly if the overall scan time and number of participants are modest (see Wall, 2023 for discussion). Consistent with the hypothesis that the MB advantage arises primarily from more volumes (Constable & Spencer, 2001; Miller et al., 2015), these improvements were largely removed when we sub-sampled only odd-numbered volumes. The only exception to this pattern was lvATL, which showed increased activation precision for MB protocols. We also found an increase in activation magnitude with multi-banding in MTL in the whole-brain analysis. The reason for these increased signal magnitudes is unclear—it could reflect an interaction between MB and ME modifications, but tests of this interaction did not reach significance.

Another known issue with increasingly high multi-band acceleration is slice leakage (Todd et al., 2016). However, we found no evidence of signal deviations (for both univariate or multivariate analyses) in areas expected to exhibit leakage for either single- or multi-echo protocols. This could be due to the fact that an acceleration factor of 2 was used in this study, where Todd and colleagues reported significant leakage with acceleration factors greater than 4.

Finally, it is interesting that we found no significant interactions between ME and MB modifications, in either the ROI or whole-brain analyses. In other words, there was no evidence that ME and MB are synergistic; their effects appear to be additive.

### 4.3 Limitations

As with many typical studies, the sample size is relatively small (especially as we had to discard participants due to poor behavioural performance) and the run length is shorter than most neuroscientific studies might implement. However, increasing the statistical power in this way is difficult if the goal is to compare multiple protocols as the full experiment needs to be repeated several times. Relatedly, this is the reason that our study design was not optimal for cross-validated multivariate analysis (in having multiple independent runs).

We showed that even modest changes to a standard protocol can lead to improvements (three echoes and MB factor of 2). We selected three echoes for the ME sequence, which is the minimum required to estimate linearity to separate signal from noise components using tedana. In theory, having a greater number of echoes would allow for better T2* estimation and allow for more accurate estimates of BOLD and non-BOLD signals (estimated using tedana); however, it would come with some trade-offs. For example, increasing in-plane acceleration (with corresponding SNR penalty), longer TR (reducing statistical power), or reduced spatial coverage. In conjunction, similar issues are likely to be experienced with very high MB accelerations, where either increases are redundant or detrimental (e.g., Wall, 2023). In principle, one can utilise more echoes to span a greater range of T2* and/or increase the MB factor to increase sampling rate (or resolution). However, to our knowledge, we are not aware of a study that empirically compares the effect of increasing echoes in task fMRI and the interaction with MB across such a large range of factors. Future work might be interested in developing an “optimal” recipe although it is likely to depend on the brain regions of interest.

Here we used a GRAPPA in-plane acceleration of 3. A lower acceleration factor of 2 might have reduced the g-factor (noise amplification associated with each voxel during acceleration), but pilot work showed that the shortest TE we could achieve with GRAPPA = 2 on our scanner was 15 ms. We wanted to achieve a TE closer to the 9–12 ms used in previous studies (Halai et al., 2014; Poser et al., 2006), so increased to GRAPPA = 3 to achieve a shortest TE of 13 ms. Nonetheless, future research could focus on optimising the trade-off between achieving a short first echo and increasing the g-factor. Indeed, future studies could explore a greater range of parameters, such as the number of TEs and their spacing in multi-echo fMRI.

A strength of the current study was to investigate both univariate and multivariate analyses in the semantic paradigm. Due to the number of protocols we compared, we did not have time to acquire multiple runs on each protocol, which would enable cross-validation of decoding for example. Nor did we explore more sophisticated decoders (such as Support Vector Machines), since our aim was not to maximise decoding performance, but simply to compare across MR sequences, though future studies could use more sophisticated, cross-validated decoding to determine whether the results generalise.

A final limitation is that our EPI volumes were tilted approximately 30° up from the AC-PC line to avoid Nyquist ghosting artefacts in the temporal lobes. Given the other parameters that we selected, in terms of number of slices and thickness, this meant that the superior parietal lobe was missing in some participants. This region is not expected to be activated in this specific task (c.f., Lambon Ralph et al., 2017, detailing the semantic network) and we have no reason to believe the protocols would behave any differently for these regions; however, future studies may want to expand the FOV for whole-brain coverage.

## 5 Conclusions

We showed that modifications to a typical fMRI protocol can lead to benefits in activation magnitude, precision, and decoding through multi-echo and/or multi-band methods. In general, we found that multi-band proved to be beneficial for activation precision (T-statistics) and multivoxel decoding across many ROIs, whereas the multi-echo was mainly beneficial in areas affected by susceptibility, and improved activation magnitude. We observed some loss in quality for multi-echo methods in more central parts of the brain, most likely owing to the in-plane acceleration (GRAPPA) required to acquire multiple echoes. Nonetheless, the MEMB protocol used here is a promising default option for fMRI on most brain regions, particularly those that suffer from susceptibility artefacts, as well as offering the potential to apply advanced post-processing methods to take advantage of the increased temporal (or spatial) resolution of MB protocols and more principled ICA-denoising based on TE dependence of BOLD signals.

## Supplementary Material

Supplementary Material

## Data Availability

Data and code have been made publicly available at https://github.com/AjayHalai/Sensitivity_of_MEMB.
