# Supplementary material for: Comparing the effect of multi-gradient echo and multi-band fMRI during a semantic task
Source: Imaging Neurosci (Camb). 2025 Dec 11;3:IMAG.a.1043. doi: 10.1162/IMAG.a.1043 (PMC13288498; doi:10.1162/IMAG.a.1043)
Supplement: Supplementary Material [file IMAG.a.1043_supp.pdf]

Supplementary Materials

Supplementary Materials 1

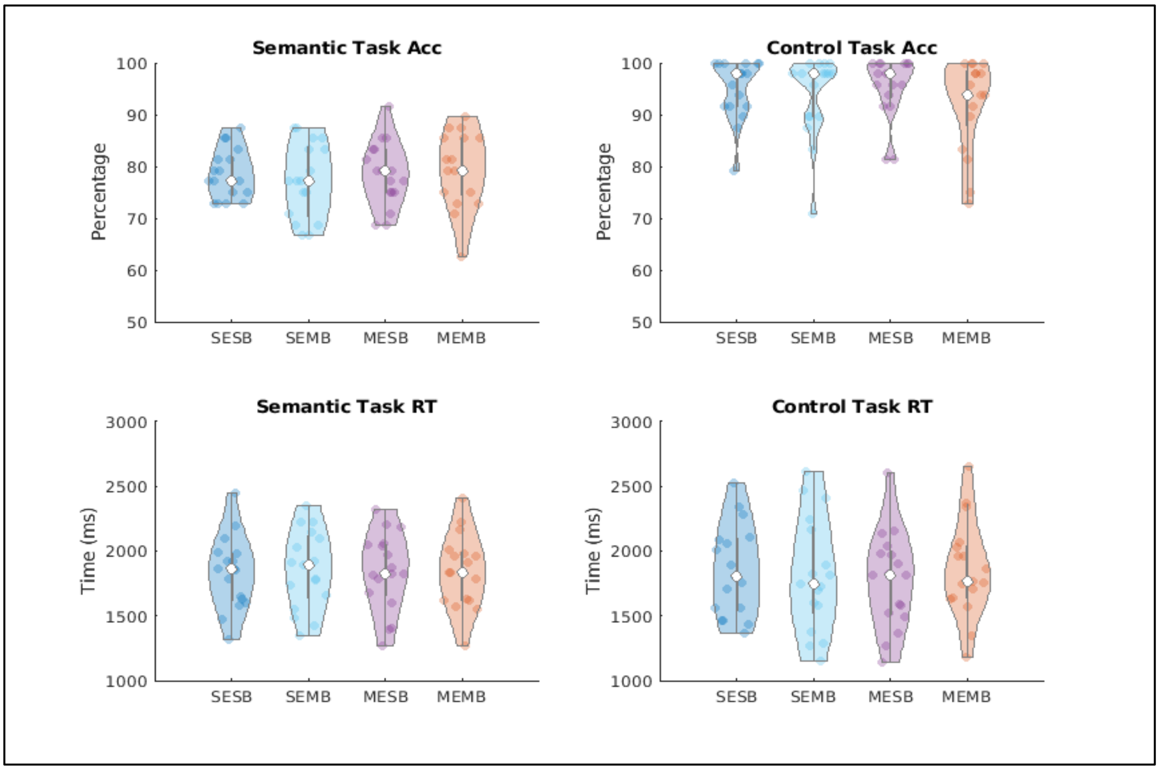

Supplementary Materials 1. Accuracy and reaction time data for semantic and control trials. Overall, the figure shows consistent behavioural performance across protocols. The top row shows lower accuracy for semantic compared to control trials, whereas reaction time did not differ. Abbreviations: Single echo single band (SESB), single echo multi band (SEMB), multi echo single band (MESB), multi echo multi band (MEMB).

Supplementary Materials 2

Table. Showing statistical comparisons across semantic network regions of interest (ROI) for activation magnitude (contrast of betas), activation precision (statistical T-values) and decoding (cosine dissimilarity). Note, ROIs are from the full semantic network identified in Humphreys et al., 2015. [positive directional contrast *p<0.05 uncorrected, **FWE corrected p<0.05 using permutation of maximal T-statistic across 36 tests; negative directional contrast †p<0.05 uncorrected, ††FWE corrected p<0.05 using permutation of maximal T-statistic across 36 tests]

Metric	Contrast	lFP	lvATL	lIFGtri	lpMTG	rITG	lmMTG	lTP	rSTG	rPCG
Activation magnitude	ME>SE	-0.79	2.14*	0.27	0.17	2.64**	-0.24	-0.21	1.22	0.82
	MB>SB	-0.31	2.75**	1.29	0.46	0.39	0.75	-0.02	-0.32	0.55
	Medn>ME	1.05	-0.52	-0.47	-0.73	-2.27†	0.55	0.32	-0.40	-0.94
	Mbodd>SB	-0.54	1.35	0.96	-0.08	0.49	0.97	0.22	-0.32	0.65
Activation precision	ME>SE	-0.85	0.36	0.40	-0.02	-0.07	0.41	0.14	1.21	1.25
	MB>SB	1.90*	5.78**	3.45**	3.62**	4.58**	0.62	0.08	-1.26	0.62
	Medn>ME	1.51	5.37**	3.21*	0.43	1.05	0.37	-0.25	-1.66	-2.33†
	Mbodd>SB	0.46	3.82**	1.12	0.36	1.01	0.73	-0.09	-0.08	0.72
Decoding	ME>SE	1.27	0.21	-1.02	-1.11	0.53	0.57	0.04	1.64	0.06
	MB>SB	-0.51	-3.76**	-3.02**	-2.88**	0.02	-1.32	-2.50*	-0.61	-1.51
	Medn>ME	1.15	-3.59**	-2.15*	-0.71	-1.78*	-1.03	0.01	-0.42	0.71
	Mbodd>SB	-0.59	-1.23	-1.75	-1.13	2.03†	-1.55	-1.85*	-0.19	-1.47

Supplementary Materials 3

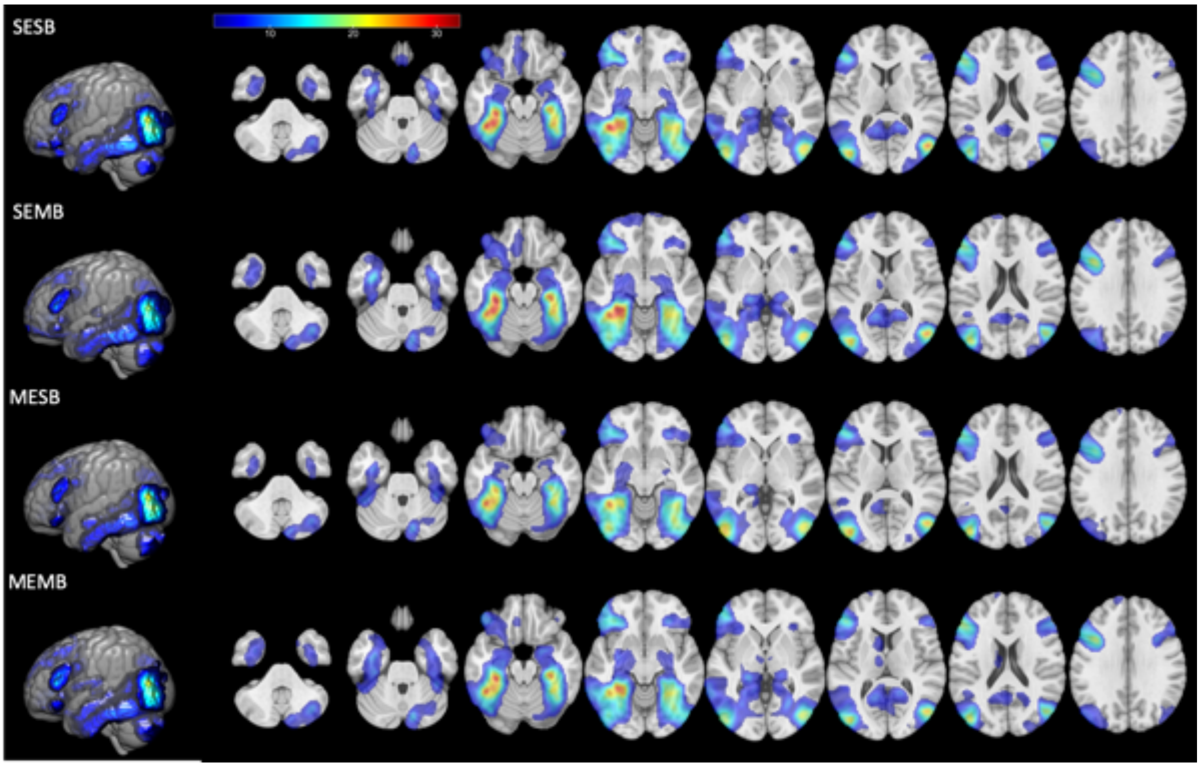

Supplementary Materials 3. Whole brain results for the Semantic>Control contrast for each protocol [t-value 3.28-32.8], thresholded at $p<0.001$ voxel height FWE-cluster corrected $p<0.05$. Abbreviations: Single echo single band (SESB), single echo multi band (SEMB), multi echo single band (MESB), multi echo multi band (MEMB).

Supplementary Materials 4

Table. Showing significant cluster and peak information for select contrasts of interest when comparing activation magnitude.

Contrast	Cluster extent (voxels)	z-value	x	y	z	Anatomy
ANOVA EOI	979	6.05	-32	-42	-8	Left lingual gyrus
		3.53	-24	-16	-22	Left Hippocampus
		3.45	-32	-26	-30	Left temporal fusiform cortex pos
	689	5.15	30	-26	-18	Right parahippocampal gyrus pos
		5.13	28	-44	-6	Right lingual gyrus
		5.08	34	-38	-12	Right temporal fusiform cortex pos
ME>SE	395	4.89	-44	-48	-32	Left temporal occipital fusiform cortex
		4.84	-50	-42	-26	Left inferior temporal gyrus post
	522	4.27	6	30	-2	Forceps minor
		4.14	-4	34	0	Left cingulate gyrus ant
		4.06	12	38	2	Forceps minor
		3.59	-16	40	4	Forceps minor
	488	3.97	24	50	14	Right frontal pole
		3.96	18	56	-2	Forceps minor
		3.82	30	46	4	Right Inferior frontal occipital fas
SE>ME	918	5.47	-34	-44	-14	Left temporal occipital fusiform cortex
		4.99	-26	-42	-6	Left lingual gyrus
		4.88	-24	-36	-12	Left parahippocampal gyrus pos
		4.52	-36	-28	-16	Left temporal fusiform cortex pos
	613	5.11	28	-26	-18	Right parahippocampal gyrus pos
		4.5	36	-42	-14	Right temporal occipital fusiform cortex
		4.45	28	-44	-4	Right lingual gyrus
		3.3	40	-52	-16	Right temporal occipital fusiform cortex
MB>SB	912	6.07	-32	-42	-8	Left lingual gyrus
		4.53	-32	-32	-22	Left temporal fusiform cortex pos
		4.11	-24	-18	-22	Left Hippocampus
	579	5.14	34	-38	-12	Right temporal fusiform cortex pos
		4.81	34	-30	-18	Right temporal fusiform cortex pos
SB>MB	N/A					

Supplementary Materials 5

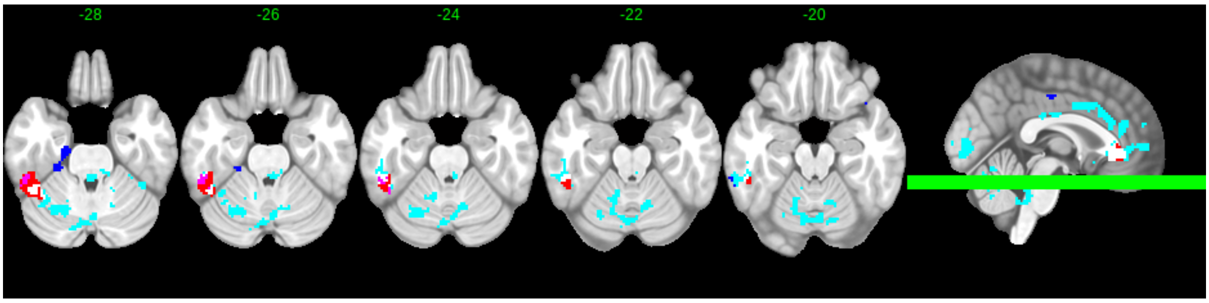

Supplementary Materials 5 showing the main effect of multi echo > single echo for data smoothed at 8 (red), 3 (blue) and 0 (green) mm FWHM. Analysis was conducted using the SnPM toolbox (N=5000 permutations), with a cluster-level inference set at cluster-size (cluster-forming threshold $p<0.001$ and significant cluster threshold $p<0.05$). We applied non-parametric tests because the unsmoothed data violates assumptions of parametric statistical tests. Note, that the overlap between 0- and 3-mm smoothing (cyan) is almost identical (hence no green colour visible) and there is some overlap between all three methods (white).

Supplementary Materials 6

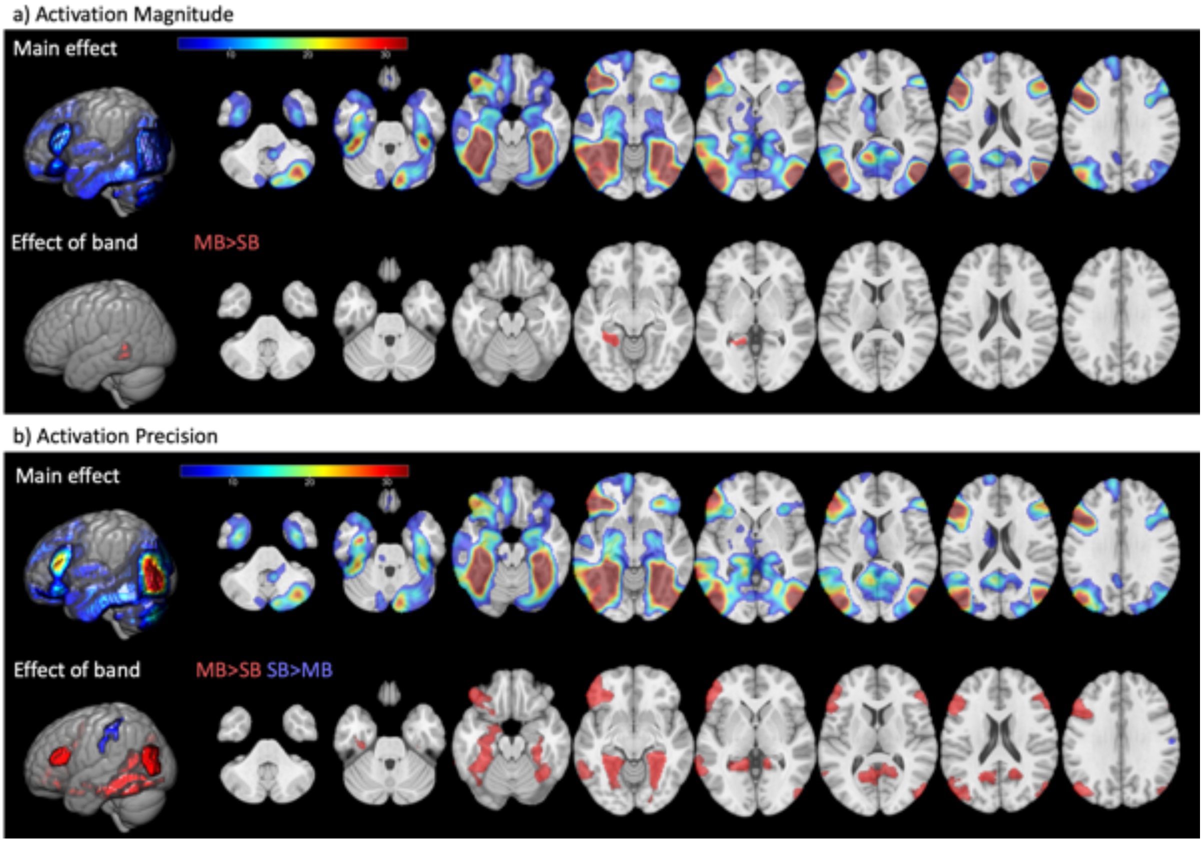

Supplementary Materials 6. Whole brain results for the effect of ICA-denoising and band (2x2 ANOVA) on the multi-echo data. 6a) Results for activation magnitude (contrast betas) and 6b) for activation precision (statistical t-values) for semantic>control. Both sections show the average effect across all protocols [t-value 3.28-32.8] and directed effects of band (MB>SB [red] and SB>MB [blue]). Note that no clusters survived correction for the main effect of ICA-denoising. Abbreviations: Multi band (MB), single band (SB).

Supplementary Materials 7

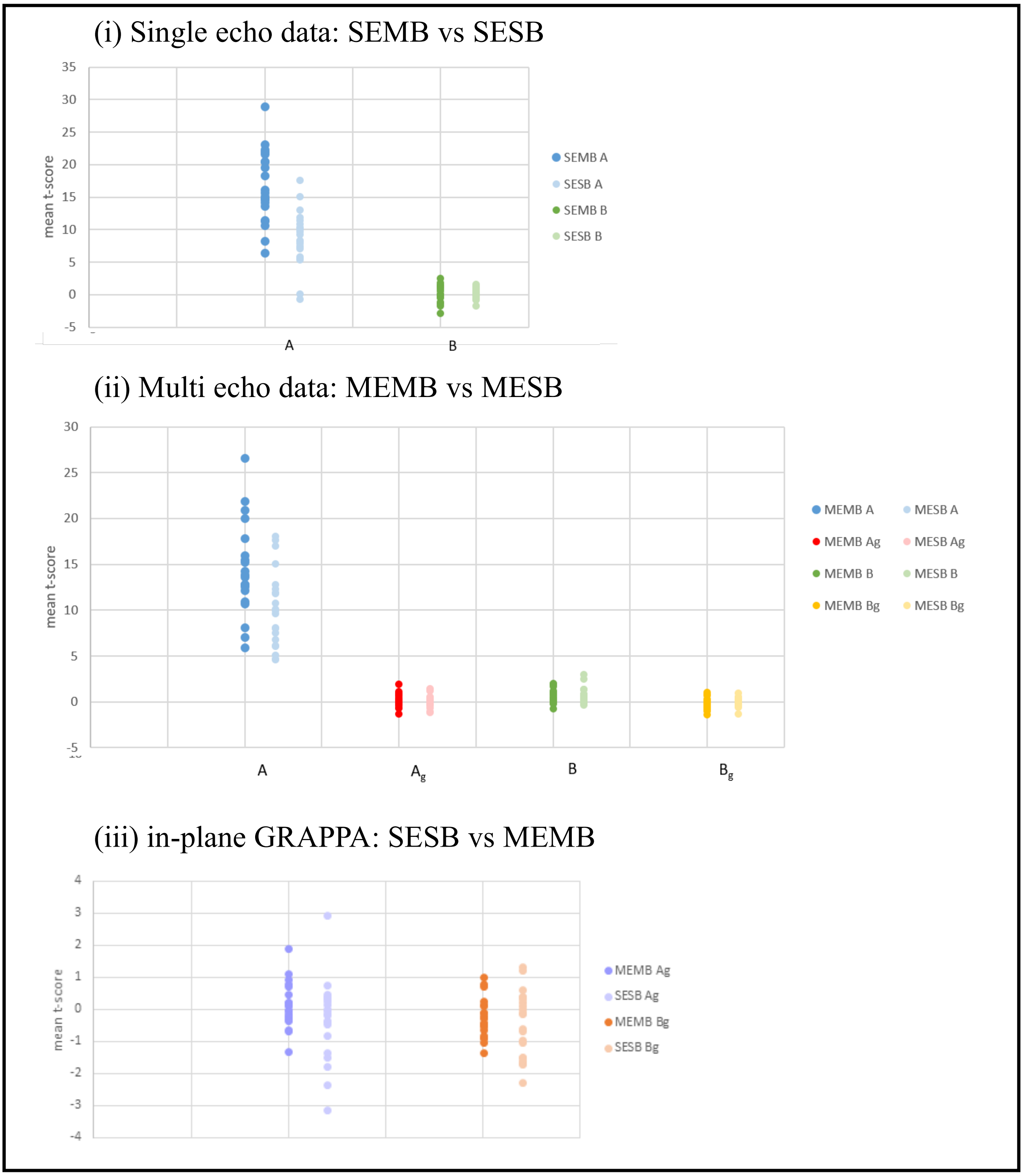

Supplementary Materials 7. Mean t-values obtained for each subject, per seed and artefact ROI. Mean t-values for (i) SEMB and SESB; (ii) MEMB and MESB; and (iii) SESB and MEMB data. Abbreviations: Single echo single band (SESB), single echo multi band (SEMB), multi echo single band (MESB), multi echo multi band (MEMB).
